# Exploring home rehabilitation therapists’ experiences of supporting older persons to physical exercise after acute hospitalization: a qualitative interview study

**DOI:** 10.1007/s41999-024-00972-5

**Published:** 2024-04-06

**Authors:** Christina Sandlund, Linda Sandberg, Sebastian Lindblom, Nathalie Frisendahl, Anne-Marie Boström, Anna-Karin Welmer

**Affiliations:** 1 Division of Physiotherapy, Department of Neurobiology, Care Sciences and Society, Karolinska Institutet, Stockholm, Sweden; 2 Academic Primary Health Care Centre, Region Stockholm, Stockholm, Sweden; 3 Division of Family Medicine and Primary Care, Department of Neurobiology, Care Sciences and Society, Karolinska Institutet, Alfred Nobels allé 23, Huddinge, 141 83 Stockholm, Sweden; 4 Department of Geriatric Medicine, Capio Geriatrik Dalen, Capio Elderly and Mobil Care, Stockholm, Sweden; 5 Theme of Women’s Health and Allied Health Professionals, Medical Unit Occupational Therapy and Physiotherapy, Karolinska University Hospital, Stockholm, Sweden; 6 Theme Inflammation and Aging, Nursing Unit Aging, Karolinska University Hospital, Huddinge, Sweden; 7 Research and Development Unit, Stockholm’S Sjukhem, Stockholm, Sweden; 8 Division of Nursing, Department of Neurobiology, Care Science and Society, Karolinska Institutet, Huddinge, Sweden; 9 Women´s Health and Allied Health Professionals Theme, Medical Unit Medical Psychology, Karolinska University Hospital, Stockholm, Sweden; 10 Aging Research Center, Department of Neurobiology, Care Sciences and Society, Karolinska Institutet and Stockholm University, Stockholm, Sweden

**Keywords:** Primary care, Rehabilitation therapy, Transitional care, Qualitative study, Exercise, Older adults

## Abstract

**Purpose:**

After hospitalization, older persons may face a decline in physical function and daily independence. In-hospital exercise interventions can mitigate this decline, and continued support from primary healthcare post-discharge may enhance sustainability. This study aimed to explore home rehabilitation therapists’ experiences of supporting physical exercise after acute hospitalization, including exercise programs initiated during hospital stay.

**Methods:**

This qualitative study was conducted alongside a randomized-controlled trial to investigate prerequisites for a transitional care intervention. Twelve interviews were conducted with physiotherapists, occupational therapists, and managers across seven rehabilitation therapy services in Stockholm, Sweden. Data were analyzed using reflexive thematic analysis.

**Results:**

The analysis generated the theme *Striving for individualized support for physical exercise, although limited resources and a fragmented home care risk to direct support away from those who need it the most*. It was based on four subthemes: *The starting point is always the patient's current needs, goals, and prerequisites*, *Continuing the exercise initiated during hospitalization by adapting it to the patient’s situation at home*, *Work premises not tailored to patients with complex care needs,* and *A home care organization that lacks coordination and unified purpose.*

**Conclusions:**

Interventions supporting older persons to physical exercise after acute hospitalization need to be tailored to the individual, support motivation, and be adapted to the patient’s home situation. Challenges may arise when care recourses lack alignment with the patients’ needs, and when the collaboration among care providers is limited. The findings contribute valuable insights for future studies incorporating transitional care interventions in similar context.

## Key summary points

**Aim:**

The aim of this study was to explore home rehabilitation therapists’ experiences of supporting physical exercise after acute hospitalization, including exercise programs initiated during hospital stay.

**Findings:**

The participants experienced that they were striving for individualized support to physical exercise, although limited resources and a fragmented home care risk to direct support away from those who need it the most.

**Message:**

Transitional care interventions need to be individualized, support motivation for exercise during hospitalization, and be adapted to the patient’s situation at home, as well as involve relevant stakeholder in the design and implementation.

## Background

Approximately half of adults aged 65 years and older experience impaired physical function following acute hospitalization [1]. After discharge, many patients do not regain the function they had before hospitalization. They lose independence, and are at risk for reduced well-being, institutionalization, readmissions, and death [1, 2]. A hospital stay is often accompanied by low levels of physical activity, which may play a major role in causing the negative health consequences associated with hospitalization [3]. There is evidence indicating that exercise interventions provided during hospital stay improve or prevent decline of physical function at discharge [4, 5]. There is, however, still uncertainty regarding how to design exercise intervention tailored to the various needs of older hospitalized patients, with or without frailty [4].

The current study was conducted alongside a three-armed randomized-controlled trial (RCT) entitled “Preventing functional decline in acutely hospitalized older patients (PREV_FUNC)”, carried out in geriatric hospitals in Stockholm, Sweden. The study protocol has been described elsewhere [6]. In brief, the aims were to (1) evaluate the effects of multicomponent exercise interventions (interventions that include both mobility and strengthening exercises) on physical function (primary outcome) at discharge from hospital and 3 months thereafter in comparison with usual care in acutely hospitalized patients aged 75 years and older, and (2) to evaluate if a comprehensive multicomponent exercise program that include several exercises is more effective than a simple multicomponent exercise program that only include walking and sit-to-stand exercises. At discharge from the geriatric hospital, patients allocated to the simple exercise program were provided with written instructions on how to perform the exercises and encouraged to continue with the program as self-training at home.

Acutely hospitalized older adults often have several concurrent diseases with a need of continued healthcare services [7]. Furthermore, there is a gradual change towards shorter length of hospital stay and an increased care of frail and ill older persons at their homes [7, 8]. One possible approach to increase the sustainability of exercise interventions introduced during hospital stay is that the intervention continues after discharge at home with support from primary healthcare. However, studies on exercise interventions in older adults with such approach are lacking [9, 10].

Transitional care has been defined as a set of actions designed to ensure the coordination and continuity of healthcare as patients transfer between different locations or different levels of care within the same location [11]. Before a possible future development of the PREV_FUNC study to include a transitional care intervention in which home rehabilitation therapy services are instructed to support the patient in continuing with exercise programs initiated in geriatric hospital care, there is a need to explore the prerequisites for such implementation. Furthermore, there is a need to understand the context of home rehabilitation therapy, in which such intervention would be implemented. According to the Medical Research Council framework for process evaluation of complex interventions [12], uncertainties related to context in which interventions are implemented and evaluated can be investigated by exploring the experiences, views, and attitudes of those who will be involved.

Thus, to better understand the context of home rehabilitation therapy for older persons and prerequisites for implementing a transitional care intervention, the present study aimed to explore home rehabilitation therapists’ experiences of supporting physical exercise after acute hospitalization, including exercise programs initiated during hospital stay.

## Methods

### Study design

We conducted a qualitative interview study alongside the PREV_FUNC study using reflexive thematic analysis [13] with an inductive and descriptive phenomenological approach [14]. The methodological orientation of the study was constructivism, which emphasizes the importance of participants' perspectives in shaping that reality, and as a researcher, you influence and shape the knowledge created through the analytical process [15]. The interviews were conducted alongside the exercise interventions and data collection of the RCT. The qualitative analysis was conducted before the analysis of RCT data. The Standards for Reporting Qualitative Research (SRQR) [16] was used for the report of the study.

### Setting and participants

The present study was conducted in Stockholm, Sweden. In Stockholm, the region is responsible to provide health- and medical care to all residents, including hospital care and primary healthcare. The primary healthcare centers are responsible for providing medical care, and nursing care (including home healthcare). The primary care rehabilitation services are independent from the primary healthcare centers and are assigned to provide home rehabilitation therapy delivered by physiotherapists, occupational therapists, and dieticians. The municipalities are responsible for providing social services (e.g., assistance with activities of daily living).

Purposive sampling was used to recruit participants working with home rehabilitation therapy either as physiotherapists, occupational therapists, or managers. To capture a variance of experiences, we strived to recruit female and male participants of different age and from different work places in different locations of Stockholm. Participation did not required experience of caring for patients included in the PREV_FUNC study. Thirteen professionals were invited to participate in the study, whereof one participant dropped out before the interview because of long-term sick leave.

The study was approved by the Swedish Ethical Review Authority (2020-06505/2022-01776-02). All study participants gave their written informed consent to participate.

### Data collection

To explore participants’ experiences, we developed a semi-structured interview guide consisting of an opening question, followed by topic questions and probing questions (Table 1). The interview guide was pre-tested with two home rehabilitation therapists outside the research group, then revised by the research group, and finally tested with a potential study participant.

**Table 1:** The semi-structured interview guide

Opening question	Tell me about your reflection on the study
Topic questions	Describe how you usually care for patients who have been discharged from acute geriatric in-patient care
	Tell me about your experiences of supporting patients to physical training after acute hospitalization, including exercise programs initiated during acute hospitalization
	Describe how you collaborate with other care providers to support patients to exercise at home
	What do you see as barriers and opportunities with implementing an intervention in were you support patients as needed to adhere to an exercise program initiated during geriatric hospital stay?
Probing questions, examples	Can you tell me more about that?
	How did you feel about that?
	What did you do then?
	What led you to that conclusion?

One week before the interview, the participants received a one-page letter with information about the PREV_FUNC study (focusing on the simple exercise as self-training at home), which they were asked to reflect upon before the interview. Individual interviews with the participants were conducted by CS who also collected background information on the participants in connection with the interviews.

During the last four interviews, new information emerged sparingly. In dialogue, LS and CS made the assessment that further interviews would not add any more themes. The interviews were conducted from August to November during 2022. They lasted for 22–57 min (in mean, 35 min), and took place during participants working time in the format they preferred: one in person, and 11 via the digital platform Teams M365. The interviews were audio recorded and transcribed verbatim.

### Data analysis

The analysis aimed to identify, analyze, and report content and meaning of patterns across data [13]. The process started with reading and re-reading of the data. Initial codes were generated by identifying relevant features of the data, and data relevant to each code were sorted systematically into potential themes. CS conducted the initial analysis, and LS repeated the procedure independently to validate and reflect on the initial themes and codes. CS and LS moved constantly back and forward between the data, codes, and themes. The following analytical process included an ongoing reflexive dialogue with the whole research group, which involved revision of themes and refining the naming of themes until consensus was reached. The analysis was conducted manually with the use of matrices to sort and compare codes and themes across data.

### Reflexivity

Reflexivity in the research group included acknowledging and critically reflecting on their experiences, perspectives, and assumptions, and how it influenced the research process [17]. The researchers came from different disciplines and clinical context: CS is a nurse, with a specialist degree in primary healthcare nursing, and with experiences of home healthcare. LS is an occupational therapist working in geriatric hospital care; SL is a physiotherapist with experience from neurologic and geriatric hospital care; NF is a physiotherapist with experiences of working in primary healthcare; AMB is a nurse and associate professor in nursing for older people with experiences in geriatric hospital care and nursing home; and AKW is a physiotherapist with experiences in geriatric hospital care and associate professor in aging research. The researchers shared their viewpoints and interpretations during the iterative analytical process. All the researchers were experienced in qualitative analysis. CS, LS, AMB, and AKW were involved in designing and evaluating the PREV_FUNC study.

## Results

Twelve participants from seven rehabilitation centers in diverse areas of Stockholm participated in the study: two occupational therapists and ten physiotherapists, whereof four also were managers. Their mean age were 47 years (range 29–59 years), 83.3% (*n* = 10) were women. In mean, they had been in their profession for 21 years (range 2–33 years), and at their current workplace for 8 years (range 0.5–29 years).

**Fig. 1:**
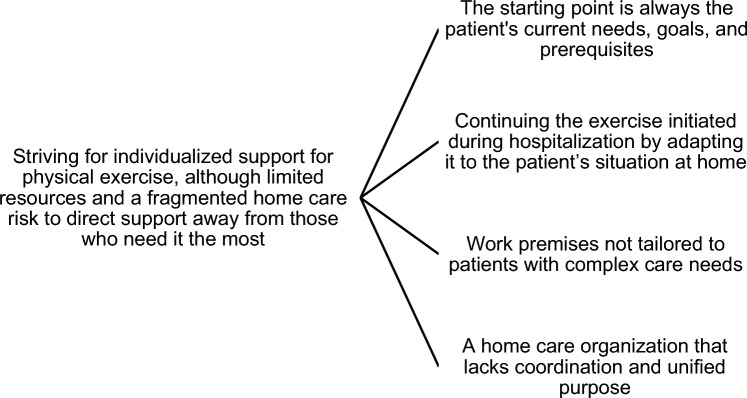
Themes of home rehabilitation therapists’ and managers experiences of supporting older persons to physical exercise after acute hospitalization, including exercise programs initiated during acute hospitalization

As shown in Fig. 1, the analysis generated the theme *Striving for individualized support for physical exercise, although limited resources and a fragmented home care risk to direct support away from those who need it the most.* The theme was based on four subthemes of participants’ experiences.

### Striving for individualized support for physical exercise, although limited resources and a fragmented home care risk to direct support away from those who need it the most

Regardless of whether exercise programs had been initiated at the hospital or if the patient received instructions for exercises to do at home, the participants stressed that the starting point is always the patient's current needs, goals, and prerequisites. They could see the benefits of laying the foundation for motivation for training already during hospital stay, and then continuing the exercise initiated during hospitalization by adapting it to the patient’s situation at home. However, they experienced work premises not tailored to patients with complex care needs, and a home care organization that lacks coordination and unified purpose.

### The starting point is always the patient's current needs, goals, and prerequisites

The participants experienced that it was uncommon that patients were provided with exercise programs to strengthen general physical functioning at discharge from the hospital, and that the referrals from the hospital seldom included information about any exercise programs. They found the sit-to-stand- and walking exercises included in the PREV_FUNC study relevant and feasible as self-training at home and in line with usual home rehabilitation. However, they stated that their starting point for exercises is always the patient's current needs, goals, and prerequisites, regardless of whether an exercise program was initiated at the hospital and whether the patient brought home any instructions.

A referral can look so different; it can be incredibly brief or quite long. We go there with a certain background but still somewhat open-minded, making our own assessment and review of the situation, and trying to create a plan together with the patient. (P1, physiotherapist, manager).

According to the participants, the first home visit focused on assessing functional ability, how the patient manages at home, the risk of falls, and the need for assistive device. They then planned their further interventions and visits based on the assessment, what function the patient would like to achieve, and the home environment. The fact that the first visit is commonly a team visit (including a physiotherapist and an occupational therapist) made the therapists aware of each other’s interventions, which facilitated continued cooperation in relation to the patient’s goal. For instance, the occupational therapist could follow up on training programs initiated by the physiotherapist, and vice versa.

Follow-up visits to confirm progress were considered as important for the patient’s motivation. They supported motivation by helping the patient to find their inner drive and personal incentives for training. When the patient had a clear goal, they worked towards it together with the patient.

Usually, you need a very clear motivation that you bring out every time before each training session. 'Come on, you're going out to meet them again. You should be able to play with your grandkids. Come on, this is to go grocery shopping on your own, go and get the milk yourself'. That's how I work, making sure they own their goals and motivation. (P6, physiotherapist).

### Continuing the exercise initiated during hospitalization by adapting it to the patient’s situation at home

The participants experienced that many patients were discharged from the hospital too early, because patients often loose functions during hospital stay, and some even returned home with worsened function.

So many ends up in the hospital and deteriorate to an extent that perhaps the hospital stay is too short. They lose many functions and, well, even their daily life, including activities and both the motivation and energy, well, I perceive that many patients quickly decline after their hospital stay. (P12, physiotherapist).

Therefore, participants highlighted a need for physical exercise to begin already during the hospital stay, which then could be continued as self-training after discharge.

If it's already been instructed and in progress, with a conversation initiated about the need to train independently, then a part of our job has already been done. I believe it would, to some extent, make things easier for us. We'd just come home more to remind, 'Do you remember that this is what you should do now?'. (P2, physiotherapist, manager).

Additionally, they found it appealing to have a treatment goal on physical functioning at the hospital that followed all the way to home. However, they could see certain difficulties in setting goals and making rehabilitation plans at the hospital that are also valid at home. A perspective that the participants highlighted was that learning a training program already at the hospital could help the patients initiate self-training early, without the delay that occurs rehabilitation therapists first must make an assessment and perhaps come back 1 or 2 weeks later with a training program.

Now, we're coming from the rehabilitation center and all, but maybe we only have one or two home visits. So, a lot is required from oneself. That's why I think it's great that the patient already gets a program from the hospital. So, they don't have to wait for us to start something, and then it needs to be followed up. And that... It takes some time. (P8, occupational therapist).

The participants emphasized that regardless of the kind of exercise program initiated at the hospital, it had to be adapted to the patient’s situation at home—the exercises the patient was able to do at the hospital may not be possible at home, and what was difficult during the hospital stay may work well in the home environment. One participant expressed it like this: *We can follow up and make it more relevant at home, because when you do it on a hospital bed, everything might feel obvious, but then when you come home, you know? (P2, physiotherapist, manager).*

Moreover, they experienced that training programs initiated at the hospital was not always anchored with the patient or adapted to the patient’s home environment. One participant said: *Many receive programs and exercises but don't know why they're taking them home, and they're not quite sure when or how to do them. (P12, occupational therapist)*. Nevertheless, they saw an advantage of the possibility to continue to support the patient on the exercise program initiated at the hospital.

### Work premises not tailored to patients with complex care needs

The participants experienced lack of time and a high workload as an obstacle to support self-training in patients who have complex needs. They said that patients who have been referred to home rehabilitation therapy after discharged from acute geriatric hospitalization often are frail, worried, and tired. Many patients have cognitive impairments, are at risk of falls, and are weak and unable to take their own initiative to perform every-day tasks. Often, they are unable to get up by themselves from the sofa or from a chair, and many are bedridden.

The participants felt sorry for that space in the calendar many times determines when and how often they could offer the patient follow-ups. According to the participants, their work situation was characterized by high workload due to many patients, a need for a large turnover of patients, and a responsibility for an economy in balance. They found it difficult to plan their work due to many new or urgent cases and that working in the patient's home offered a tough work environment. They felt there are too many home care organizations to communicate with, and often large distances between the patients, which both were time-consuming. Moreover, they felt insufficient because of staff shortage, mainly related to difficulties in recruiting new colleagues. These aspects affected their ability to provide sufficient support to patients with complex care needs.

The participants described how they had to prioritize regarding the compensation system, which favors home visits or digital visits, but not phone calls, and how they had to prioritize what in their specific care assignment. Moreover, they needed to prioritize their time and resources and assess every patient's needs in relation to those of other patients.

I can't say how often we can support an older person after acute hospitalization. It's those who are out there who must make that assessment. And it's in relation to other cases that come in. If it's fractures or trauma patients, they might need several times a week, so it's always an ongoing assessment. (P3, physiotherapist, manager).

The participants thought that if home care rehabilitation therapy should participate in a transitional care intervention involving them in supporting exercise programs initiated during geriatric hospital stay, it is of importance to include patients with motivation and ability to exercise and take own responsibility for their exercise. This was because they had limited possibilities for frequent and long-term follow-ups. They experienced that they do not have enough resources to continuously support self-training in patients who lack an inner drive to exercise on their own and who lack self-care abilities.

Then I try a couple of times, like once a week, I go there, and I see that nothing has happened yet. The person doesn't seem interested at all. Then I feel like I can spend my time on someone who needs and wants to make a change. (P7, physiotherapist).

### A home care organization that lacks coordination and unified purpose

The participants experienced that the many various home care services involved in the patient's care and concern at home are working in parallel tracks. They experienced a lack of coordination, and that they do not know what they can expect from each other. The participants missed the feeling that they all are working towards the same goal. The fact that different home care services (i.e., home healthcare, home rehabilitation therapy, and home care services by the municipalities) have different assignments often lead to a lack of understanding, and a misunderstanding of each other’s mission and possibilities. One participant said: *I feel that the municipality is a vacuum on its own, and we are on our own. It should have been one and the same, working together so that we can allocate these budgets together for individuals. (P7, physiotherapist).* Moreover, the participants experienced that all care providers want to form their own opinion and decide for themselves which interventions are needed, based on their mission, and that everyone has different areas of knowledge and wears different glasses when they assess needs and plan interventions.

Home rehabilitation therapy services operates under one agreement, and hospitals operate under other agreements, and there isn't always mutual understanding. Somewhere, there needs to be coordination in this. We need to speak the same language and have the same ambition so that we don't burden each other and have assumptions about how things should be done. (P2, physiotherapist, manager).

The participants saw the social service provided by the municipalities as their most important collaborator when supporting patients to self-training. However, they had lowered their expectations of what the social services could do when it comes to supporting the patient's functional ability. They said that the managers or staff from social services had made it clear to them that it is not their mission to support patients to practice physical exercise, and experienced that the staff generally lacks time and an understanding of a rehabilitative approach.

The social service staff is so pressed for time that they almost take them (the older persons) by the arms and put them in a wheelchair. Then I say, 'Well, the patient can walk with a walker; that's exercise.' They just don't have the time to wait or take the time needed to bring out the walker. (P4, physiotherapist).

When it comes to collaboration with the primary healthcare centers, they said that it seldom was time for communication if they, for instance, met a nurse at the patient’s home. Moreover, they were seldom invited by the primary healthcare centers to participate in their team meetings that aims to reach agreement on how the care providers involved in the patient’s care could come together to meet the patients’ needs for care and assistance. They had no experiences of initiating such meetings themselves.

The participants said that they were cautious about involving relatives in supporting the patient to training, because they did not want to lay the responsibility on them. They also experienced that the patient often did not want them to involve their relatives in such support. One participant expressed it like this: *Some relatives are very involved and supportive and have no problem with it. But for some, they are very clear about it: 'No, that's not…' It's very important to just be a relative. (P8, occupational therapist).*

## Discussion

This study explored the context of home rehabilitation therapy for older persons, including prerequisites for future implementation of a transitional care intervention involving physical exercise. The theme *Striving for individualized support for physical exercise, although limited resources and a fragmented home care risk to direct support away from those who need it the most* illuminates’ therapists and managers experiences of supporting physical exercise after acute hospitalization, including exercise programs initiated during hospital stay.

In this study, the participants experienced that *the starting point is always the patient's current needs, goals, and prerequisites*, regardless of whether exercise programs were initiated at the hospital or if the patient received instructions for home. However, they could see benefits of laying the foundation for motivation for training already during hospitalization, and then *continuing the exercise initiated during hospitalization by adapting it to the patient’s situation at home*. This result indicates that there is a functioning transitional care between geriatric in-patient care and home rehabilitation therapy services regarding exercise interventions. However, not in the sense that the rehabilitation therapist follows what is initiated during in-patient care. Instead, they plan interventions and support based on the patient's needs and situation at home, as well as on the patient's own goals for training. Such person-centered care [18] may be specifically important in home-based care to empower older persons to feel in control and truly at home in their own space [19].

Moreover, participants could experience that patients were discharged from the hospital too early when many returned home with worsened function. This finding aligns with the findings of previous studies showing that approximately one-third of older persons lose independence in activities of daily living (ADL) following acute hospitalization [2, 20]. Fear of leaving the hospital can contribute to the progression of disease, attributed to unresolved physical symptoms, and worry about being left alone [21].

Receiving rehabilitation therapy after acute hospital care can prevent unplanned healthcare utilization and hospital readmissions 3 months after hospital discharge [22]. The sit-to-stand- and walking exercise intervention included in the PREV_FUNC study was adopted from a previous RCT conducted in acute geriatric in-patient care in Spain [23]. The intervention may be relevant to test as a transitional care intervention, since it was experienced as acceptable by patients during hospitalization (results of a feasibility study of the PREV_FUNC study, submitted manuscript), and as relevant and feasible as self-training at home by participants in the present study. However, the participants stressed that regardless of intervention, it must be anchored with the patient. This finding aligns with those of a previous study showing that frail older persons chose physical exercises based on their personal goals, and that they need information on how to perform it safely in relation to their prerequisites [24].

Participants in the current study experienced *work premises not tailored to patients with complex care needs*. Notably, a majority of older persons receiving rehabilitation therapy following acute hospitalization have multimorbidity, cognitive impairments, a need for assistance in ADL, and previous experiences of fall [22], which was also experienced by the participants in the present study. However, they had limited possibilities to support self-training in patients who lack motivation and ability to take an own responsibility for their training. The finding aligns with the previous studies in the context of home rehabilitation therapy and other home care services indicating that resourceful patients often get priority when care resources are scarce [25], and that persons with limited self-care abilities and cognitive impairments are deprioritized because of limited time and resources, limited use of memory aids, and limited rehabilitation potential [18, 26–28]. The conflict between prioritizing resources and the patient's needs may pose ethical challenges in the provision of person-centered quality home care, compromising the principle of justice [25, 29].

Participants in the present study experienced *a home care organization that lacks coordination and unified purpose*. This result mirror the cross-cultural challenges that can appear between care organizations with distinct values and priorities and highlight the need for healthcare providers to learn about each other's roles and functions [30]. Lack of communication between professionals in different levels of care could create skewed conceptions, misunderstandings, and mistrust regarding each other's work [31], resulting in challenges for communication and information transfer, which in turn lead to inconsistent and inadequate delivery of care [32]. However, collaboration among healthcare providers seems to be crucial when implementing transitional care interventions for older adults [11]. For instance, it enhances the patient's ability to adapt to the home situation after an acute hospitalization by strengthening the patient's motivation and ability to take initiative [30].

Prior research has identified factors that support the care of frail older persons with multimorbidity. These facilitating factors seems to remain consistent across perspectives, whether from healthcare providers, patients, or relatives, and included communication, information, coordinated care, a person responsible for care planning, and resources distributed in relation to the patient’s actual care needs [33–35]. Similarly, a qualitative study investigating how multidisciplinary teams in various contexts (general practice, hospitals, and community services) facilitate successful transitions of care revealed that familiarity with the patient and each other’s mission, and bridging gaps in the system were crucial for overcoming challenges. However, because of high workload and limited resources, they only made certain efforts to address these challenges when providing care to patients with especially complex transitional care needs [36]. Previous studies, as well as the current study, reveal that there is a potential for improvements regarding common goals and collaboration between hospital and home care organizations for the care of older frail persons. To be successful in transitional care research, the described situation will require more collaborative study designs where stakeholders from all involved care organizations are involved during the research process.

By addressing the following elements, we aimed to establish trustworthiness of the study, strengthening the validity and reliability of the findings. First, we used purposeful sampling to ensure diversity in participants, considering variations in professional affiliation, roles within their units, work experiences, and gender. This approach enhances the credibility of findings, enabling a thorough exploration of experiences, and attitudes among professionals involved in providing home rehabilitation therapy to older persons after acute hospitalization [37]. The sample specificity, the narrow study aim and the good interview dialogue align with the concept of information power [38]. The information power of the present study, as well as the detailed description of the study context, participant characteristics, and the process of data collection and analysis, allows readers to evaluate the transferability of our findings to similar settings. Second, the preliminary interview guide was pre-tested outside the research group in two steps. This process of developing the semi-structured interview guide contributes to the objectivity and trustworthiness of the study and makes the results more plausible [39]. Thirdly, the researchers maintained an ongoing reflexive dialogue throughout the study, acknowledging personal biases and preconceptions. The researchers’ reflexive dialogue along with the description of researcher’s background allows readers to consider the potential influence of researchers' perspectives on the findings [17]. Member checking was not employed, which could be seen as a limitation of the study. However, the evidence that member checking improve qualitative research findings is limited [40].

## Conclusions

This study identified both opportunities and obstacles for implementing a transitional care intervention involving home rehabilitation therapy services in supporting the older patient to continue with an exercise program introduced during acute hospitalization. Opportunities included benefits of laying the foundation for motivation of training already during hospitalization, and then continuing the rehabilitation process by making the training relevant to the patient’s situation at home. Challenges included limited care resources and ethical dilemmas when care is not adapted for patients with significant healthcare needs, as well as difficulties in collaborating to meet the patient's needs. This study highlights the importance of involving relevant stakeholders, such as patients, relatives, and care providers in the design and development of the intervention. Moreover, the results may help interpret upcoming results of the 3-month follow-up of the PREV_FUNC study.

## Data Availability

The data that support the findings from this study are not publicly available due to the permission given from the Swedish Ethical Review Authority.
